# Nuclear Phospholipids and Signaling: An Update of the Story

**DOI:** 10.3390/cells13080713

**Published:** 2024-04-19

**Authors:** Irene Casalin, Eleonora Ceneri, Stefano Ratti, Lucia Manzoli, Lucio Cocco, Matilde Y. Follo

**Affiliations:** Cellular Signaling Laboratory, Department of Biomedical and Neuromotor Sciences, University of Bologna, 40126 Bologna, Italy; irene.casalin2@unibo.it (I.C.); eleonora.ceneri2@unibo.it (E.C.); stefano.ratti@unibo.it (S.R.); lucia.manzoli@unibo.it (L.M.); matilde.follo@unibo.it (M.Y.F.)

**Keywords:** nucleus, phosphoinositides, phospholipases C, myelodysplastic neoplasms (MDS), muscle, brain

## Abstract

In the last three decades, the presence of phospholipids in the nucleus has been shown and thoroughly investigated. A considerable amount of interest has been raised about nuclear inositol lipids, mainly because of their role in signaling acting. Here, we review the main issues of nuclear phospholipid localization and the role of nuclear inositol lipids and their related enzymes in cellular signaling, both in physiological and pathological conditions.

## 1. Introduction

Even though the presence of phospholipids in the nucleus has been reported since the 1960s [1], it has been difficult to assign a role to these molecules in the nucleus.

In the sixties, it was shown that the phospholipid content of active chromatin was five-fold higher compared to repressed chromatin [2]. Few laboratories were interested in pursuing the biological significance of this observation with regards to cell cycle regulation, but the obstacle was the persisting controversy over the extent to which these phospholipids might arise by contamination from non-nuclear membranes. Manzoli and Cocco investigated this problem, and in 1977, they concluded that non-contaminating phospholipids were genuinely associated with non-histone chromosomal proteins (NCHP), perhaps regulating DNA stability and replication. They also reported that NHCP fractions prepared from leukemic B-lymphocytes contained 40% less sphingomyelin and four-fold higher levels of phosphatidylcholine, compared to normal B-cells [1]. The clear demonstration that lipids are really present at the chromatin level and that they do not derive from the nuclear membrane was given from Albi et al. [3] by using the radioiodination of the fatty acids technique. This seemed to be a good way to overcome the persisting controversy in order to investigate if there could be a role for nuclear phospholipids. Indeed, in 1980, the analysis of Triton-treated nuclear material to remove nuclear membranes was described [4]. This short paper presented an important finding that refuted the criticism that phospholipids in nuclear preparations must have arisen from contaminating membranes. Indeed, data had shown nuclear phospholipids need not even be associated with membranes. But still, some skepticism remained. That was the time of the emergence of inositol lipids as cellular signals [5]. They are present at very low levels and at that time were canonical plasma membrane signals. However, because of a hint by Smith and Wells [6], it was worthwhile to investigate the presence and possible role of these peculiar phospholipids in the nucleus. In 1983, Smith and Wells had concluded inositol lipid synthesis occurred in the nuclear envelope, probably contaminated by the endoplasmic reticulum. But it was shown that membrane-free nuclei were able to synthesize phosphatidyl 4,5 bisphosphate (PtdIns(4,5)P2) in a cytoplasm-independent manner [7]. Several follow-up papers appeared that helped the nuclear inositol lipid idea become more generally accepted, and then others also began to publish studies into nuclear phosphoinositide signaling [8,9]. In 1992, a nuclear-specific species of Phospholipase PLCβ that was activated within 2 min treatment of 3T3 mouse fibroblasts with insulin-like growth factor-1 was identified [8].

## 2. Nuclear Phosphoinositides and Nuclear Functions

Polyphosphoinositides (PPIns) constitute a group of phospholipids originating from the primary compound Phosphatidylinositol (PtdIns). PtdIns consists of a myo-inositol hydrophilic head group linked to a hydrophobic diacylglycerol (DAG) tail backbone through a phosphodiester bond. The hydroxyl groups of the myoinositol head can be reversibly phosphorylated at the 3, 4 or 5 position yielding to seven distinct PPIns variants: PI3P, PI4P, PI5P, PI(3,4)P2, PI(3,5)P2, PI(4,5)P2 and PI(3,4,5)P3 [9].

As mentioned in the introductory paragraph, the nuclear presence of all phosphoinositides but one, PI(3,5)P2, was reported in the 1960s. Since then, biochemical studies ensured that the nuclear pool of PPIns was not a consequence of cytoplasmatic pool contamination and that it was regulated distinctly from the latter, while further studies defined nuclear targets potentially interacting with nuclear PPIns defining downstream signaling pathways.

Despite the fact that PPIs are amphiphilic, meaning they have a polar inositol head group that faces toward the cytoplasm/nucleoplasm and non-polar hydrophobic fatty acid tails embedded in the lipid bilayer [10], 40% of nuclear phosphoinositides can exist in a non-membrane state [11], mainly in nuclear speckles [12], membrane-less nuclear domains enriched in pre-mRNA splicing factors, located in the interchromatin regions of the nucleoplasm of mammalian cells [13]. Hydrophobic lipids probably exist within the aqueous nucleoplasm through the binding with nuclear proteins, but the exact mechanism and the specific proteins remain largely unclear. Steroidogenic factor-1 (SF-1; NR5A1 in the official nomenclature), a nuclear receptor transcription factor that plays a crucial role in the regulation of adrenal and gonadal development, function and maintenance [14], is one candidate nuclear protein binding PPIs. The research group guided by Blind showed, in 2014, that in an x-ray crystal structure of PI(4,5)P2 bound to the SF-1 ligand-binding domain, the hydrophobic acyl chains of PI(4,5)P2 are hidden deep in the hydrophobic core of the SF-1 protein, while the hydrophilic phosphoinositide headgroup is solvent-exposed [15]. Additionally, in 2023, they demonstrated the first evidence of the association between SF-1 and nuclear PI(4,5)P2 in fixed human cells. They established two isogenic HEK cell lines with a single, stably integrated tetracycline-inducible 3X-FLAG-tagged wild-type/mutated SF-1 and, after tetracycline treatment to induce SF-1 expression, they analyzed the nuclear accumulation of the immunofluorescence signal from antibodies directed against the headgroup of PI(4,5)P2. The results showed that the ectopic expression of wild-type SF-1 in HEK cells associates with the induction of a nuclear signal that cross-reacts with PI(4,5)P2 antibodies [16]. Further studies should analyze how SF-1 initially acquires the PI(4,5)P2 phospholipid and whether PIP2 antibody is directly recognizing PI(4,5)P2 bound to SF-1, or if the signal is an undiscovered, indirect byproduct of SF-1 expression. 

Nuclear PPIs have several functions encompassing DNA synthesis, epigenetic signaling, transcription factor regulation and DNA damage signaling [17]. 

Concerning DNA synthesis, in 1988, the research group of Cocco obtained two different clones of Swiss 3T3 cells, one unresponsive and one responsive to the mitogenic stimulus by insulin growth factor 1 (IGF1). They illustrated that when highly purified nuclei from responsive cells treated with IGF1 were exposed to [γ- 32 P]adenosine triphosphate, there was a decrease in PIP and PIP2 labeling compared to the controls, alongside the activation of Protein Kinase C (PKC). On the contrary, they did not notice the transient effect of PPIs nor the PKC activation in unresponsive cells. These results show a direct link between polyphosphoinositide metabolism, nuclear PKC and early events leading to cell division [18]. 

PPIs can participate in epigenetic and transcription signaling through histone modification regulation and transcription complex interaction. An example can be represented by PI5P binding to several nuclear proteins through plant homeodomains (PHD) that in turn interact with methylated and non-methylated lysine residues in histone tails and regulate gene expression [19]. Within the basal transcription complex Direct Transactivator-Transcription Factor IID (TFIID), there exists a component known as Transcription initiation factor TFIID subunit 3 (TAF3). TAF3 plays a crucial role in governing both embryonic stem cell pluripotency and myogenic differentiation. It possesses a PHD finger that interacts with Histone 3 Lysine 4 trimethylation (H3K4me3), thereby regulating gene expression [20]. This enables changes in nuclear PI5P to impact on myogenic differentiation. Under non-differentiating conditions, Phosphatidylinositol 5-phosphate 4-kinase type-2β (PIP4K2β), a nuclear-localized lipid kinase, phosphorylates PI5P to PI(4,5)P2 [21], thereby attenuating the expression of genes required for myogenic differentiation induced by TAF3. Upon induction of differentiation, PIP4K2β is localized into the cytoplasm, leading to increased nuclear PI5P [22].

PPIs are finally involved in DNA damage repair as well. Phosphatidylinositol-4-phosphate 5-kinase (PIP5K) is a PI(4,5)P2 synthetizing enzyme [23] which interacts with the p53 tumor suppressor protein, whose post-transcriptional expression is reduced by the knockdown or inhibited activity of Phosphatidylinositol-4-Phosphate 5-Kinase Type 1 Alpha (PIP5K1A) [24]. The p53 also directly interacts with phosphatidylinositol 4,5-bisphosphate (PIP2) inducing an interplay between p53 and the heat shock protein 27 (HSP27), leading to p53 stabilization [25]. Additionally, the PIP2-p53 complex is a substrate for Inositol Polyphosphate Multikinase (IPMK), which generates p53 bound to PI(3,4,5)P3, leading to the activation of nuclear Protein Kinase B (PKB) that in turn associates nucleophosmin (NPM/B23) to regulate the expression of genes involved in DNA repair and cell survival in response to a DNA damage signal [26]. PKB is one of the most well-studied targets of PI(3,4,5)P3, with the interaction occurring through the Pleckstrin Homology domain (PH domain) of PKB. Moreover, biochemical assays found that also nucleophosmin is a nuclear target of PI(3,4,5)P3, and that the interaction of B23 with PI(3,4,5)P3 is required for interaction with PKB in the nucleus [27]. 

What still remains to be investigated in this context is how the nuclear PPIns pool is established and how their associated modulating enzymes and downstream signaling pathways are controlled and maintained. Are PPIs formed in the cytoplasm and then translocated to the nucleus, or are they directly synthesized in the nucleus? 

## 3. Nuclear PI-PLCs

Phosphoinositide-Specific Phospholipase C (PI-PLC) is a crucial family of enzymes encompassing 13 isoforms, further categorized into six families that exhibit varying expression patterns across different tissues: PI-PLCβ (1–4), PI-PLCγ (1–2), PI-PLCδ (1,3,4), PI-PLCε, PI-PLCζ e PI-PLCη (1–2) [28,29]. Structurally, these isoforms possess a catalytic domain with X–Y split, along with a regulatory domain comprising the C2 domain, EF-hand motif, and PH domain, all of which are highly conserved [30]. However, each isoform displays a unique combination of these domains, resulting in distinct regulatory mechanisms, functions, and tissue distributions [30]. Broadly, these enzymes act as catalysts in the hydrolysis of PIP2 into inositol 1,4,5-trisphosphate (IP3) and DAG in response to various stimuli, including growth factors, hormones, and neurotransmitters [28,29,30,31,32]. IP3 and DAG function as second messengers, with IP3 facilitating intracellular calcium ion accumulation, and both IP3 and DAG collectively activating PKC, thereby intricately regulating the cell cycle at multiple levels [28].

The cellular distribution of PLC enzymes varies based on the isoform and can change under diverse circumstances [33].

Observations on Swiss 3T3 mouse fibroblasts revealed that PI-PLC activity is activated within the nucleus independently of plasma membrane phosphoinositide metabolism. This activation, triggered by IGF-1, resulted in changes in nuclear phosphatidylinositol levels and the translocation of PKC into the nucleus [18,34]. Subsequent studies identified PI-PLCβ1 as the enzyme responsible for this nuclear activity. Additionally, PI-PLCβ1, along with other enzymes such as diacylglycerol kinase ζ (DGK ζ) and phosphatidylinositol phosphate kinase α (PIPKα), was found to localize to nuclear speckles. These findings provide insights into the distinct roles of PI-PLC isoforms within the nucleus and their involvement in cellular signaling pathways [35].

The PI-PLCβ1 enzyme, encoded by a gene located on the short arm of chromosome 20 (20p12), is characterized by two splicing variants that differ at the C-terminal domain: PI-PLCβ1a (150 kDa) and PI-PLCβ1b (140 kDa) [36]. Both isoforms contain a nuclear localization sequence (NLS) at the 3′ end, but PI-PLCβ1a also features a nuclear export sequence (NES) downstream of the NLS sequence [37]. Consequently, PI-PLCβ1a localizes in both the nucleus and the cytoplasm, whereas PI-PLCβ1b is predominantly distributed in the nucleus, particularly in nuclear regions called speckles, where gene expression regulation occurs [38]. Researchers have shown that phosphorylation of PI-PLCβ1 by P42-Mitogen-Activated Protein Kinase (MAPK) enhances its nuclear activity, but subsequent phosphorylation by PKC deactivates PI-PLCβ1 [17]. Following activation, nuclear PI-PLCβ1 plays a role in regulating the cell cycle at both the G1/S transition and G2/M progression through various molecules. Furthermore, nuclear PI-PLCβ1 has been linked to processes such as hematopoietic, osteogenic, myogenic, and adipogenic differentiation [39].

PI-PLCγ1 has been identified within the nucleus, with its Src Homology 3 (SH3) domain reported to function as a guanine nucleotide exchange factor for specific nuclear GTPases, including phosphoinositide 3-kinase enhancer and dynamin-1. Notably, the assistance provided by PI-PLCγ1 to phosphoinositide 3-kinase enhancer is independent of its phospholipase activity, influencing various cellular functions such as proliferation and survival [40]. 

Also, the PI-PLCδ family members exhibit bidirectional movement between the nucleus and cytoplasm, suggesting their potential involvement in regulating cell growth. Although PI-PLCδ1 is typically located in the cytoplasm of quiescent cells, it localizes inside nuclear structures at the G1/S boundary during the cell cycle. This isoform possesses both nuclear export and import sequences, facilitating its movement between the cytoplasm and nucleus [40].

Another variant, PI-PLCδ4, demonstrates nuclear localization in various cellular contexts, including regenerating rat liver, serum-stimulated Swiss 3T3 cells, AH794 rat ascites hepatoma cells, and src-transformed 3Y1 cells [41]. Upon stimulation by mitogens, PI-PLCδ4 expression is upregulated in the nucleus, where it plays a significant role in promoting cell growth and is among the early genes activated during the transition from G1 to S phase in the cell cycle [42]. Specifically, the nuclear δ4 isoform shows a substantial increase at the G1 to S phase transition and maintains high levels until the end of the M phase. While some studies suggest that this isoform is exclusive to the nucleus, others have not replicated this finding [41]. Additionally, PI-PLCδ4 has been recently found to mediate the epidermal growth factor (EGF)-induced nuclear Ca^2+^ signaling and downstream events. Indeed, EGF induces hydrolysis of nuclear PI(4,5)P_2_, essential for the Ca^2+^ release from the nucleoplasmic reticulum by inositol 1,4,5-trisphosphate receptors, by the intranuclear PI-PLCδ4. PI-PLCδ4 may consequently pave the way to new therapeutic targets to modulate the proliferative effects of EGF [43].

Given the emerging roles of these molecules within the nucleus, it is intriguing to explore the involvement of nuclear PLCs in various pathologies, potentially serving as promising new markers or therapeutic targets. The following sections will specifically focus on PI-PLCβ1, but it has to be underlined it is not the unique nuclear PI-PLC involved in pathologies. An example is represented by the nuclear PI-PLCγ1 involvement in oral squamous cell carcinoma, the proliferation and tumor growth of which can be suppressed through PI-PLCγ1 signaling inhibition by p120 [44].

## 4. Nuclear PI-PLCs in Myelodysplastic Neoplasms (MDS)

PI-PLCs signaling has emerged as a significant factor in both normal and malignant hematopoiesis, as evidenced by several studies [45,46,47]. The regulatory mechanisms governing PI-PLCβ1 enzyme are linked to the pathogenesis of myelodysplastic syndromes (MDS) [48].

MDS are a heterogenous group of myeloid neoplasms depending on an alternate function of hematopoietic stem cells (HSCs), leading to ineffective hematopoiesis, dysplasia and peripheral blood cytopenia and characterized by an increased risk of evolution to Acute Myeloid Leukemia (AML) in approximately one-third of cases [49]. The International Prognostic Scoring System (IPSS) and its revised version (IPSS-R) have served as pivotal tools for predicting outcomes and guiding treatment decisions in leukemia based on initial laboratory and cytogenetic assessments [50]. Recently, advancements in next-generation sequencing (NGS) techniques have led to the creation of the Molecular International Prognostic Scoring System (IPSS-M), which classifies patients into six prognostic categories, ranked by their estimated survival and risk of leukemia progression: Very Low, Low, Moderate Low, Moderate High, High, and Very High [51]. Although allogeneic stem cell transplantation remains the only treatment opportunity for a limited fraction of MDS patients, epigenetic therapy, which include the use of hypomethylating agents (HMAs) such as Azacytidine, is the first-line approach for high-risk MDS patients [52]. 

**Figure 1:**
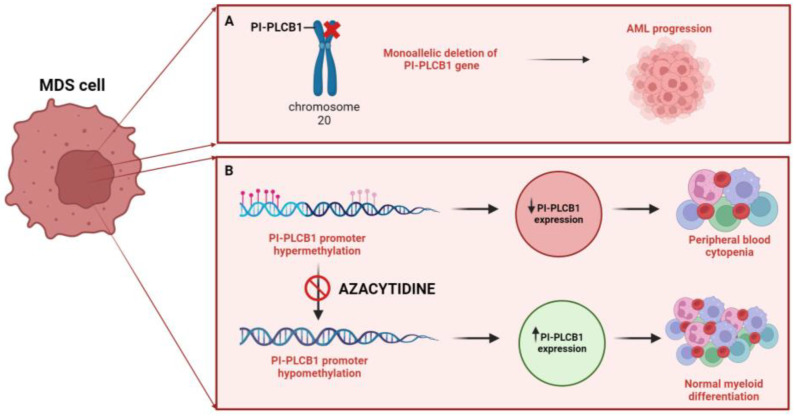
Role of nuclear PI-PLCβ1 in Myelodysplastic Neoplasms (MDS). (**A**) Correlation between increased risk of AML progression in MDS patients with the mono-allelic deletion of the PI-PLCB1 gene (del20p). (**B**) Increased expression of nuclear PI-PLCβ1, induced by Azacytidine sensitivity due to hypermethylation of the PI-PLCB1 promoter, promotes normal myeloid differentiation in MDS cells. MDS: Myelodysplastic Neoplasms; AML: Acute Myeloid Leukemia; PI-PLCB1: phosphoinositide-specific phospholipase C β1. [Created by Biorender.com].

Abnormalities in phosphoinositide-dependent signaling, epigenetic regulators, apoptosis, and cytokine interactions within the bone marrow microenvironment are defining features that contribute to the pathogenesis of diseases and neoplastic growth of MDS [53] (Figure 1). Among the enzymes of the nuclear PI cycle, nuclear PI-PLCβ1 plays a fundamental role in the regulation of hematopoietic differentiation, that seems to be related to the recruitment of Myeloid zinc finger-1 (MZF-1) [28], and suggests that this enzyme has a significant impact on the initiation and progression from MDS to AML by affecting both genetic and epigenetic processes [54,55,56]. Through fluorescence in situ hybridization (FISH), an analysis of 80 MDS cases unveiled that 43.75% of patients displayed a monoallelic deletion in the *PLCB1* gene situated on chromosome 20p12 [57,58]. In contrast, the *PLCB4* gene, encoding another signaling molecule and positioned on 20p12.3 within 1Mb of *PLCB1*, remained unaffected, even in MDS patients exhibiting the deletion of the *PLCB1* gene, suggesting a distinct and interstitial deletion pattern for *PLCB1* [57,58]. Interestingly, it has been demonstrated that the monoallelic deletion of *PLCB1* gene correlates with adverse clinical outcomes and an increased risk of AML development both in high- and low-risk MDS patients [58].

At the epigenetic level, high-risk MDS often exhibits hypermethylation of the CpG islands within the promoter region of the PI-PLCβ1 gene, as indicated in a review by Cocco et al. [30]. Additionally, Follo et al. (2009) [59] demonstrated that nuclear *PLCB1* serves as a direct target of Azacytidine, with *PLCB1* expression potentially predicting the efficacy of Azacytidine treatment. They observed that responsive MDS patients displayed higher levels of *PLCB1* during clinical improvement, as well as increased expression levels of Cyclin D3, one of the molecular targets of PI-PLCβ1, a key regulator of G1/S cell cycle phase and hematopoietic differentiation [60]. Furthermore, their study revealed a correlation between *PLCB1* levels and activated *AKT* levels, suggesting opposite roles for PI-PLCβ1 and Akt [61]. In a subsequent study [59], the analysis of *PLCB1* expression in 18 high-risk MDS patients undergoing Azacytidine treatment showed that changes in gene expression preceded clinical improvements or worsening by about two months. 

Recent studies have highlighted the crucial role of the Phosphoinositide 3-kinase (PI3K)/Akt signaling pathway in various physiological processes and its persistent activation in high-risk MDS [45]. Elevated levels of phosphorylated Akt have been observed in MDS patients’ bone marrow and peripheral blood cells, suggesting its involvement in leukemia progression [62]. Additionally, there is a notable increase in mTOR pathway activity in high-risk MDS patients, with rapamycin showing potential therapeutic effects, suggesting the therapeutic potential of targeting the PI3K/Akt/mTOR network in both AML and high-risk MDS cases [45].

These findings indicate a potential association between PI-PLCβ1 gene silencing and Akt activation in high-risk MDS, suggesting a role in disease progression towards AML. This connection may be influenced by PIP2 levels, crucial for both PI-PLCβ1 and PI3K/Akt axis activation [45]. Nevertheless, these findings are significant for high-risk MDS patients and the interplay between PI3K/Akt/mTOR and PI-PLCβ1 signaling pathways suggests potential combined therapeutic approaches to target disease progression.

## 5. Nuclear PI-PLCs in Muscle Diseases

Nuclear PI-PLCs play a crucial role also in muscle diseases by impairing the correct myogenic differentiation, in which PI-PLCs have an important role.

For studying physiological myogenic differentiation, multiple studies have explored the PI-PLCβ1-dependent signaling pathways during myogenic differentiation employing C2C12 murine myoblasts. These cells serve as a reliable and widely used model for investigating myogenesis, as they demonstrate the capacity to differentiate into myotubes under low serum conditions, allowing for the study of numerous muscle-specific genes and proteins [63]. C2C12 differentiation is characterized by a marked increase in PI-PLCβ1 and cyclin D3 expression [64,65]. Indeed, at the onset of the differentiation process, myoblast determination protein 1 (MyoD) induces cyclin D3 expression, which in turn binds to the unphosphorylated retinoblastoma protein, leading to the withdrawal of myoblasts from the cell cycle [66]. In addition, it was shown that PI-PLCβ1 could activate cyclin D3 promoter during the differentiation of myoblasts to myotubes, only in the presence of the binding of the transcription factor c-jun to cyclin D3 promoter [67,68]. Another crucial player in myogenic differentiation is IPMK, which leads to the production of more phosphorylated inositol species starting from the IP3 substrate, the PI-PLCβ1 second messenger. IPMK production of IP5 is able to induce the Wnt/β-catenin pathway, leading to the translocation of β-catenin to the nucleus where it induces downstream gene expression, among which c-jun which in turn activates cyclin D3 promoter, demonstrating IPMK acts on the same cyclin D3 promoter region targeted by PI-PLCβ1 [60]. In summary, the model proposed for myogenic differentiation suggests that PI-PLCβ1 induction triggers the hydrolysis of PIP2, resulting in the production of IP3. Subsequently, IP3 undergoes phosphorylation by IPMK, leading to the generation of increasingly phosphorylated inositol species such as IP4, IP5, and IP6. The accumulation of IP5 prompts the translocation of beta-catenin to the nucleus, where it activates c-jun, ultimately stimulating the cyclin D3 promoter.

**Figure 2:**
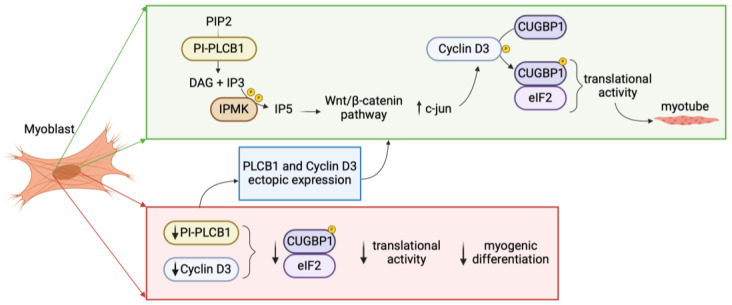
Role of nuclear PI-PLCβ1 in physiological myogenic differentiation and in myotonic dystrophies (DM). In physiological myogenic differentiation, PI-PLCβ1 metabolizes PIP2 leading to IP3 formation, that once phosphorylated by IPMK activates the Wnt/β catenin pathway. β catenin activates c-jun which in turn activates the cyclin D3 promoter. Cyclin D3-Cdk4/6 regulates the phosphorylation of CUGBP1 enabling its interaction with eIF2 activating translational activity. The downregulation of PI-PLCβ1 and consequent decrease in cyclin D3 expression leads to decreased CUGBP1-eIF2 interaction culminating in dysregulated myogenic differentiation. PIP2: phosphatidylinositol 4,5-bisphosphate; PI-PLCB1: phosphoinositide-specific phospholipase C β1; DAG: diacylglycerol; IP3: inositol 1,4,5-trisphosphate; IP5: inositol pentakisphosphate; IPMK: inositol polyphosphate multikinase; CUGBP1: CUG-binding protein 1; eIF2: eukaryotic initiation factor 2. [Created by Biorender.com].

The most studied muscle diseases with impaired PI-PLCs expression level, specifically PI-PLCβ1, are myotonic dystrophies (DM), the most common muscular dystrophies in adults [69]. They are autosomal dominant progressive multisystem disorders, which can indeed affect other non-skeletal muscle organs such as the heart, brain and gastrointestinal system. DM is divided in two subtypes: DM type 1 and type 2, both dominantly inherited with significant overlap in clinical manifestations such as myotonia, muscle weakness, insulin resistance, cardiac conduction defects, cataracts, cognitive dysfunction, and mental retardation. They are instead distinguished by the pathological origin: DM1 is triggered by the pathological expansion of a CTG triplet repeat in the gene coding for dystrophia myotonica-protein kinase (DMPK), while DM2 by a CCTG tetranucleotide repeat expansion in the ZNF9 gene, which encodes a CCHC-type zinc-finger protein [70]. These expansions alternate the signaling pathway downstream the CUG triplet repeat RNA-binding protein 1 (CUGBP1), which plays a central role in alternative splicing of specific target genes and can interact with eukaryotic initiation factor 2 (eIF2) and cyclin D3 inducing normal myogenic differentiation. In normal myotubes, cyclin D3-cyclin dependent kinase 4/6 (cdk4/6) mediate the phosphorylation of CUGBP1, increasing the interactions of CUGBP1 with eIF2 enabling translational function during normal myogenesis [71]. In DM-differentiating cells, we assist with multiple alterations leading to ineffective myogenesis: CUGBP1-eIF2 interactions are reduced and both PI-PLCβ1 and cyclin D3 expression are decreased [72]. Figure 2 shows physiological and pathological myogenic differentiation.

Interestingly, the research group of Cocco demonstrated, by transfecting myoblasts from patients with DM1 and DM2, how forced PI-PLCβ1 expression can cause a cyclin D3 expression increase, determining a partially restored phenotype of the myotubes [72]. Additionally, Salisbury E. et al., showed that the ectopic expression of cyclin D3 corrects differentiation of DM1 myoblasts through the activation of RNA CUG-binding protein, CUGBP1 [73]. 

To conclude this overview on DM, both PI-PLCβ1 and cyclin D3 could be investigated for future possible molecular therapies to induce a correct skeletal muscle differentiation in DM. 

## 6. Nuclear PI-PLCs in Neurological Diseases

The healthy human brain comprises around 60% lipids, mainly found in myelin and white matter. Phospholipids (PLs) are the most abundant lipid species, crucial for neuronal membrane structure and function. Changes in phospholipid composition can disrupt membrane fluidity, impacting cellular signaling and leading to pathological conditions, including cancer. However, the mechanisms behind phospholipid alterations in tumors are not fully understood [69]. PI-PLCs are essential components in the intricate machinery of various brain regions, exerting influence over a spectrum of neural functions and implicated in the pathogenesis of numerous brain disorders [69]. 

Among the different isoforms, PI-PLCβs stand out as the prominent ones [74], with PI-PLCβ1 as the most extensively studied, at both the cytoplasmatic and nuclear level, in brain contexts [75]. Its prevalence is notably observed in key regions like the hippocampus and cerebral cortex, as well as in specific neuronal populations such as cerebellar interneurons and telencephalic principal neurons [76]. Furthermore, PI-PLCδ isoforms, which possess both nuclear export and import sequences [77], including PI-PLCδ3 and PI-PLCδ4, exhibit distinct roles within neural architecture. PI-PLCδ3, primarily localized in the brain, contributes significantly to the radial migration of neurons during cerebral cortex development [30,78]. Meanwhile, PI-PLCδ4’s expression in both brain tissues and regenerating areas underscores its involvement not only in neural function but also in processes related to tissue repair [79].

PI-PLCβ1 has been identified as a crucial enzyme influencing various brain processes. For instance, nuclear PI-PLCβ1 (i.e., PI-PLCβ1b) is involved in the modulation of endocannabinoid neuronal excitability through DAG synthesis and can be linked to depolarization and receptor activation, maintaining the brain inhibitory pathways via 2-Arachidonoylglycerol (2-AG) [69]. Moreover, the PI-PLCβ1 pathway, which is activated via metabotropic glutamate receptors (mGluRs), is also implicated in the development of normal cortical circuit and in activity-dependent development of the cerebral cortex [80,81]. These suggest that imbalances in PI-PLCβ1 could be associated with several brain disorders, including epilepsy, schizophrenia, and neuro-oncological pathologies [79,82,83].

In addition, research has revealed that PI-PLCβ1 exhibits co-localization and physical interaction with Lamin B1, a crucial component of the nuclear lamina [84]. These findings gain significance in the context of Autosomal Dominant Leukodystrophy (ADLD), a remarkably rare and fatal neurodegenerative disorder characterized by the overexpression of Lamin B1 (*LMNB1*) due to genetic anomalies such as *LMNB1* gene duplications or deletions. Recent investigations emphasize the pivotal role played by cellular signaling and morphological changes, particularly in astrocytic function, in the progression of ADLD [85]. Given the established role of PI-PLCβ1 in various cellular processes within the brain, further exploration is warranted to ascertain potential correlations between its interactions with Lamin B1 and the pathogenesis of this ADLD. 

**Figure 3:**
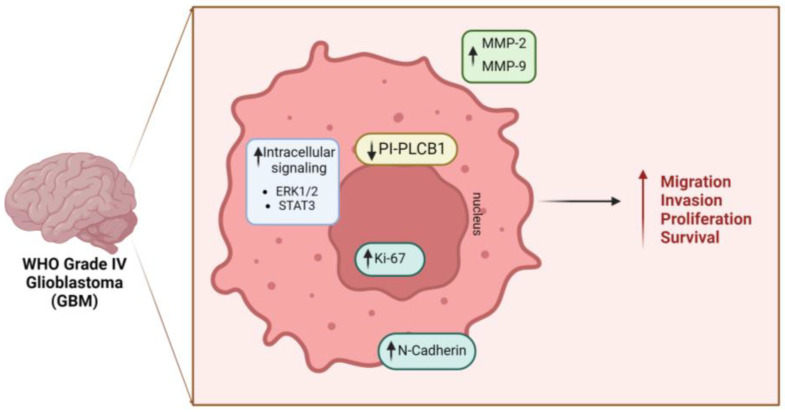
Role of nuclear PI-PLCβ1 in Glioblastoma (GBM). The downregulation of PI-PLCβ1 dictates various significant physio-pathological alterations, culminating in enhanced cellular migratory, invasive capacities, proliferation, and survival, which lead to a more aggressive phenotype. GBM: Glioblastoma Multiforme; PI-PLCB1: phosphoinositide-specific phospholipase C β1; MMP: matrix metalloproteinase; STAT3: Signal Transducer and Activator of Transcription 3 Signal Transducer and Activator of Transcription 3; ERK: Extracellular Signal-Regulated Kinase [Created by Biorender.com].

Notably, there is substantial evidence suggesting the involvement of PI metabolism in the development of Glioblastoma Multiforme (GBM), highlighting the intricate interplay between PI-PLCs and other mediators regulating cell proliferation, differentiation, migration, and growth [86,87,88] (Figure 3). GBM represents a grade IV astrocytoma, distinguished by its diverse cellular composition, genetic instability, extensive infiltration, angiogenesis, and resilience against existing treatments [89]. GBM can arise either spontaneously or progress from a pre-existing astrocytoma, both scenarios carrying a bleak prognosis. Despite ongoing efforts, current therapies for GBM exhibit limited efficacy, with only a small fraction of patients—approximately 3–5%—surviving beyond three years [90].

Analyses of the Cancer Genome Atlas and Gene Expression Omnibus have shed light on the potential significance of PI-PLCβ1 as a biomarker in high-grade gliomas (HGG). Notably, an inverse relationship between PI-PLCβ1 expression and the pathological grade of gliomas has been elucidated, suggesting its potential as a prognostic indicator and a novel signature gene for proneural (PN) subtypes within the molecular classification of HGG. Additionally, insights from Kaplan-Meier survival analysis from the Repository for Molecular Brain Neoplasia Data (REMBRANDT) underscore a correlation between PI-PLCβ1 expression and prolonged patient survival [88].

More recently, Ratti et al., not only corroborated these data, but also demonstrated that in vitro downregulation of *PLCB1* is associated with increased expression of mesenchymal markers (e.g., Slug, N-Cadherin), matrix metalloproteinases MMP-2 and MMP-9, and the nuclear marker Ki-67. Furthermore, there was an overexpression of the active form of B-catenin and heightened activation of the Extracellular Signal-Regulated Kinase (ERK1/2) pathway. Taken together, these findings indicate that silencing of *PLCB1* correlates with increased invasion, migration, and cell proliferation [86]. Indeed, after silencing of *PLCB1*, increased activation of the Stat3 pathway, a well-established oncogenic transcription factor known to play a pivotal role in tumor resistance and aggressive cancer progression in glioblastoma [86] was also observed, thereby strengthening the hypothesis that downregulation of PI-PLCβ1 in glioblastoma promotes a more aggressive phenotype. The mechanisms behind PI-PLCβ1 downregulation in high-grade tumors, particularly in glioblastoma, remain unclear. Understanding these processes and their specific roles in gliomas could lead to the discovery of diagnostic and prognostic biomarkers. Further research is necessary to identify epigenetic anomalies associated with this condition, which could inform the development of targeted therapies [86].

## 7. Conclusions

**Figure 4:**
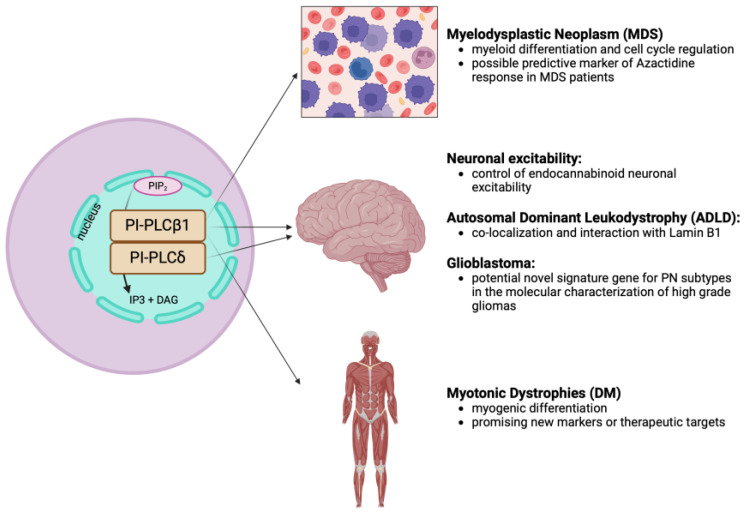
Schematic representation of the role of nuclear PI-PLCβ1 and PI-PLCδ in different diseases. PI-PLCβ1: phosphoinositide-specific phospholipase C β1; PI-PLCδ: phosphoinositide-specific phospholipase C δ; IP3: inositol triphosphate; DAG: diacylglycerol. [Created by Biorender.com].

In conclusion, this review has shed light on the significant role of nuclear PIs and nuclear PI-PLCs, particularly in hematological neoplasms, muscular diseases, and neurological disorders, including brain neoplasms. The intricate interplay of these signaling molecules in regulating cellular functions underscores their relevance in disease pathogenesis and their potential role as new markers or therapeutic targets, aiming at mitigating diseases burden and improving clinical outcome. Figure 4 provides a comprehensive overview, highlighting the involvement of nuclear PI-PLCs in different human diseases.

## Data Availability

No new data were created or analyzed in this study. Data sharing is not applicable to this article.
